# Supplementary material for: Gliadin Nanoparticles Containing Doxorubicin Hydrochloride: Characterization and Cytotoxicity
Source: Pharmaceutics. 2023 Jan 4;15(1):180. doi: 10.3390/pharmaceutics15010180 (PMC9860592; doi:10.3390/pharmaceutics15010180)
Supplement: Supplementary file 1 [file pharmaceutics-15-00180-s001.zip › pharmaceutics-2097518-supplementary.pdf]

pharmaceutics

MDPI

Supplementary Materials: Gliadin nanoparticles containing doxorubicin hydrochloride: characterization and cytotoxicity

Silvia Voci, Agnese Gagliardi, Nicola Ambrosio, Maria Cristina Salvatici, Massimo Fresta and Donato Cosco

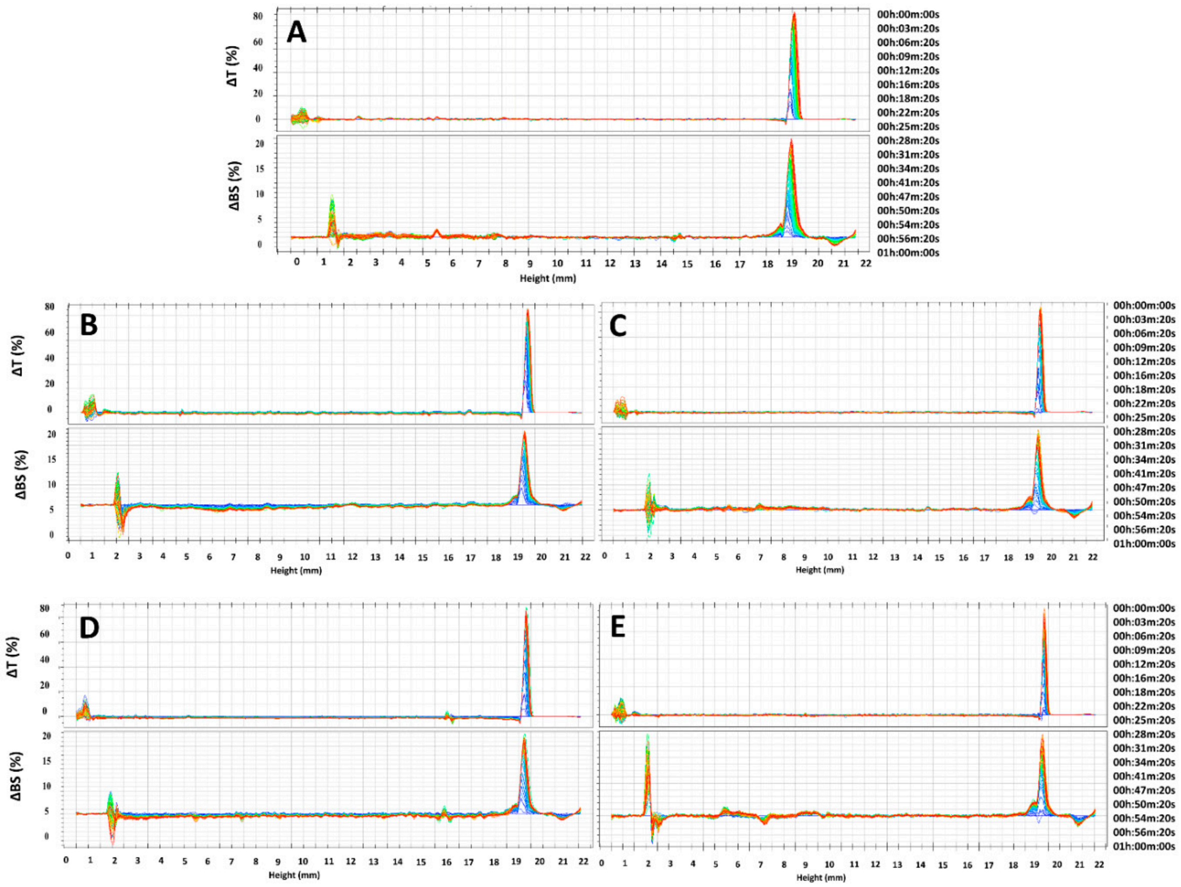

Figure S1. ΔT and ΔBS profiles of (A) empty GNPs and (B-E) DOX-loaded GNPs as a function of the drug concentration and incubation time. (B) 0.2 mg/mL; (C) 0.4 mg/mL; (D) 0.6 mg/mL and (E) 0.8 mg/mL. The analysis was performed at 25 °C for one hour.

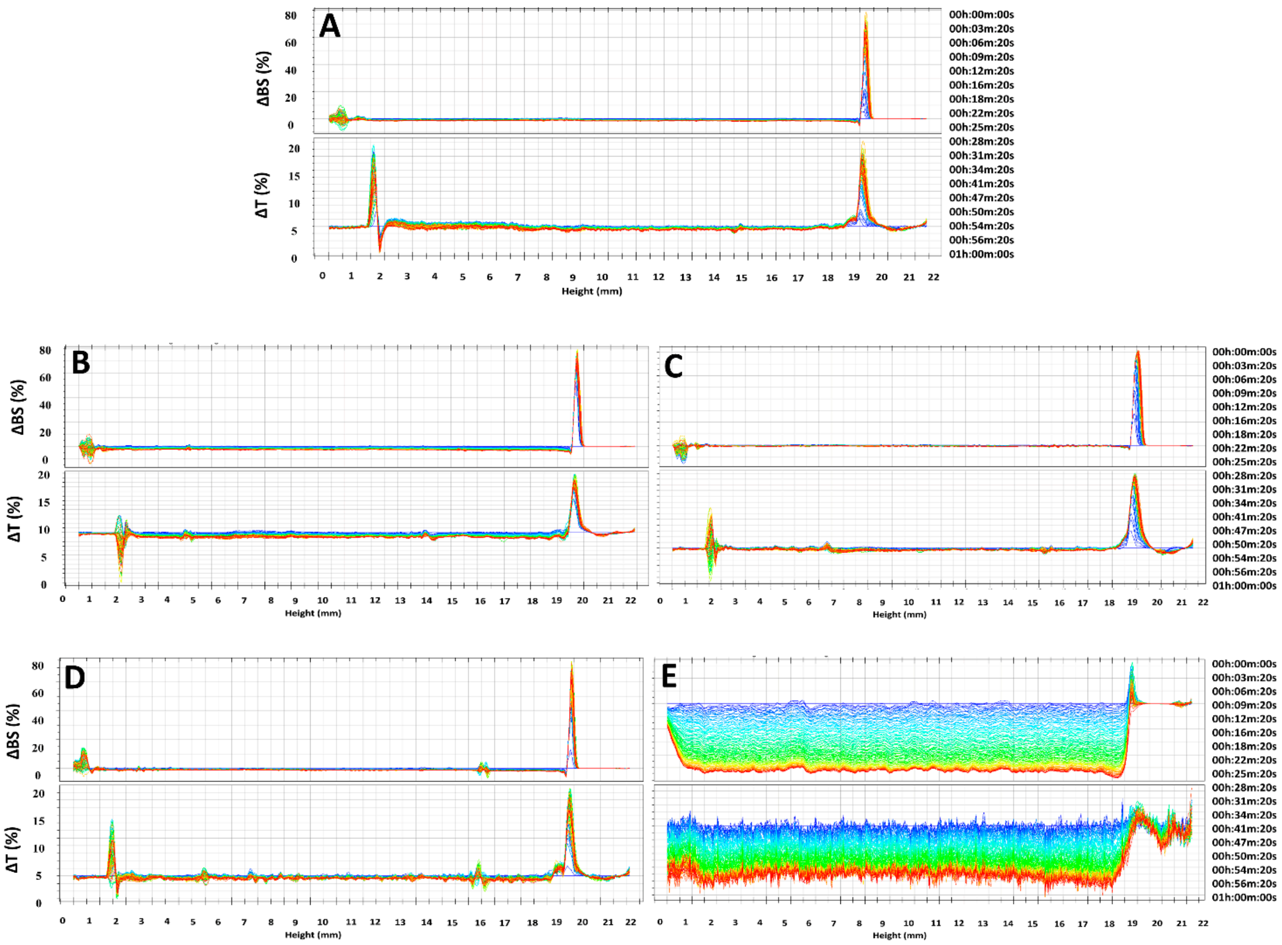

Figure S2. ΔT and ΔBS profiles of (A) empty GNPs and (B-E) DOX-loaded GNPs as a function of the drug concentration and incubation time. (B) 0.2 mg/mL; (C) 0.4 mg/mL; (D) 0.6 mg/mL and (E) 0.8 mg/mL. The analysis was performed at 37 °C for one hour.

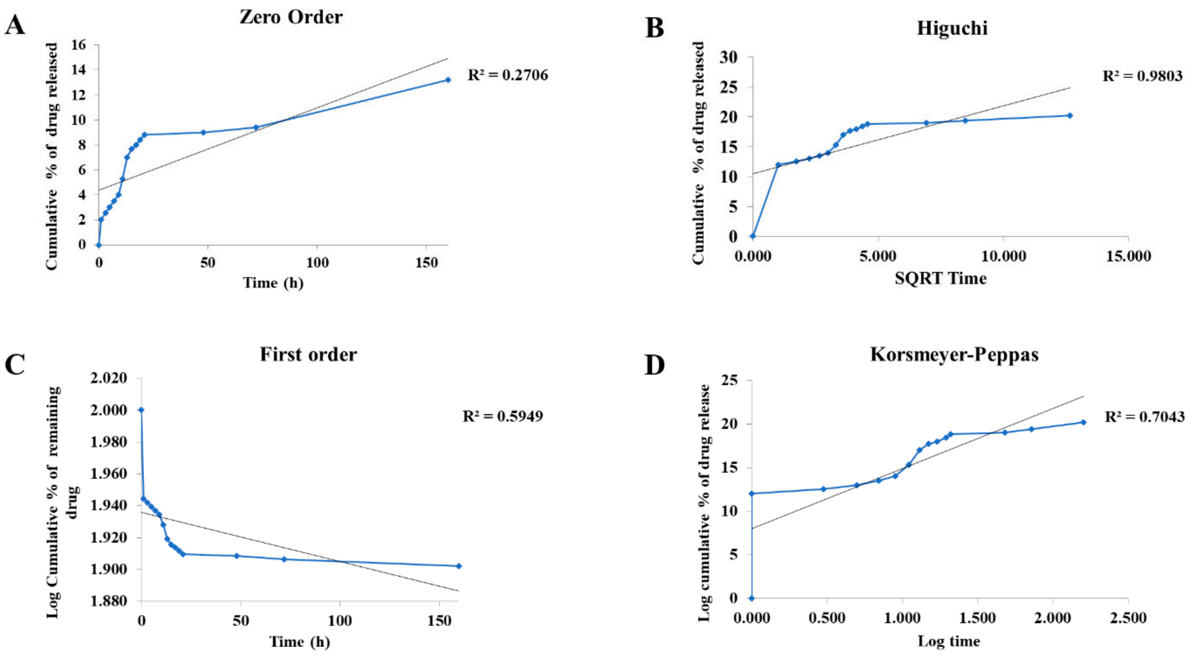

Figure S3. Drug release kinetics of DOX from DOX-loaded GNPs (600 µg/mL of drug) evaluated in physiological conditions and fitted against (**A**) Zero order, (**B**) Higuchi, (**C**) First order and (**D**) Korsmeyer-Peppas mathematical models. SQRT: Square root of time.

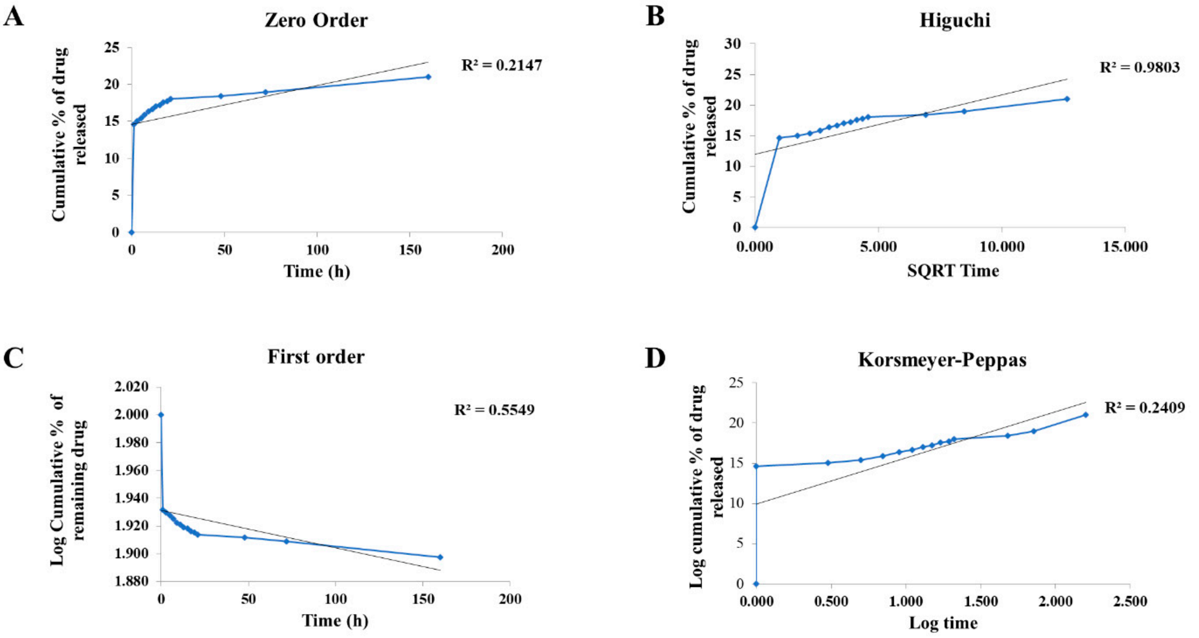

Figure S4. Drug release kinetics of DOX from DOX-loaded GNPs (600 µg/mL of drug) evaluated in simulated tumor conditions and fitted against (**A**) Zero order, (**B**) Higuchi, (**C**) First order and (**D**) Korsmeyer-Peppas mathematical models. SQRT: Square root of time.
